# Treatment experience of people living with HIV who transitioned from oral to long-acting injectable antiretrovirals in Puerto Rico: A qualitative study

**DOI:** 10.1371/journal.pone.0332661

**Published:** 2025-09-15

**Authors:** Robert A. Velez, Georgina Silva-Suarez, Kalumi Ayala, Yarelis Alvarado, Felix Badillo

**Affiliations:** 1 Pharmacy Practice Department, Nova Southeastern University, Barry and Judy Silverman College of Pharmacy, San Juan, Puerto Rico; 2 Sociobehavioral and Administrative Pharmacy Department, Nova Southeastern University, Barry and Judy Silverman College of Pharmacy, San Juan, Puerto Rico; PLOS: Public Library of Science, UNITED KINGDOM OF GREAT BRITAIN AND NORTHERN IRELAND

## Abstract

This study explores the treatment experience of people living with HIV who transitioned from oral ART to long-acting injectable therapy Cabotegravir/Rilpivirine (LAI-CAB/RPV) in Puerto Rico. Interpretative Phenomenological Analysis was used to explore the lived experiences of people with HIV 21 years old or older who transitioned from oral ART to LAI-CAB/RPV. Purposeful sampling was used to recruit study participants. Ten in-depth interviews were conducted at the clinic, audio recorded, and transcribed verbatim. Participants’ mean age was 67 years (±9), most were men (70%), and most identified themselves as heterosexual (60%). On average, they have taken oral ART for 23.2 years ± 8.7. The transition to LAI-CAB/RPV marked a profound positive change; for them, injectable ART means “freedom,” improving their quality of life, providing relief, and a sense of “normalcy.” Adherence remained robust, marked by consistent appointment attendance. They overwhelmingly encouraged others to embrace the transition, highlighting its transformative impact on their lives. Study participants’ positive experiences underscore the potential of LAI-CAB/RPV to revolutionize the standard of care, enhancing both the treatment experience and the overall well-being of people living with HIV. Continued research is crucial to refine and expand the benefits of this treatment approach.

## Introduction

Antiretroviral therapy (ART) has revolutionized the management of HIV, transforming it from a fatal disease into a chronic condition. By 2022, an estimated 39 million individuals worldwide were living with HIV [1]. Over 16,000 people are living with HIV in Puerto Rico in 2022, yet new cases have decreased over 45% from 2013 to 2022 [2]. This decline in new infections can be attributed, in part, to the significant advancements in ART and its broader accessibility. ART has played a crucial role in reducing the viral load in people living with HIV, making them less likely to transmit the virus to others [3]. Daily oral ART adherence is a critical aspect of managing HIV, yet it is often hindered by various barriers. Stigma and discrimination surrounding HIV status remain significant obstacles to adherence [4]. Individuals living with HIV may fear disclosing their status, leading to social isolation and reluctance to take their medication consistently. In addition, complex pill burden regimens and the potential for medication side effects can deter adherence [5]. The inconvenience and adverse effects of daily oral ART may discourage individuals from maintaining their treatment, which is essential for achieving viral suppression and reducing the risk of transmission [6].

In 2017, Puerto Rico was ranked 6^th^ in the prevalence of adults and adolescents living with HIV among the United States and its territories. Based on surveillance data from 2014, about 68% of people living with HIV on the island were in HIV care, but only 55% were virally suppressed and about 9% were undiagnosed [7]. Currently, collaborative agreements exist between the public and private sector as well as non-profit entities for the prevention and treatment of HIV. The Puerto Rico Ryan White Part B and AIDS Drugs Assistance Program Providers Network consists of 55 centers distributed throughout eight health regions [8]. Access to medications exists for patients without health insurance or with limited coverage through these programs. Main risk factors for HIV infection in Puerto Rico include intravenous drug abuse, high risk heterosexual sex, and men who have sex with men [7]. The greatest prevalence is in the metropolitan area of San Juan. Limitations in HIV care in Puerto Rico include stigma, barriers for prevention, inadequate education, and social determinants including housing instability and transportation [9].

The transition from oral ART to long-acting injectable therapy with Cabotegravir/Rilpivirine (LAI-CAB/RPV) represents a promising approach to addressing these challenges associated with adherence to oral ART. This approach involves the use of long-acting injectable formulations of Cabotegravir and Rilpivirine, which can maintain exposure at plasma concentrations exceeding in vitro 90% inhibitory concentrations with monthly intramuscular injections [10]. To this day, clinical trials have demonstrated that long-acting ART is non-inferior to daily oral ART, showing rates of viral suppression, treatment failure, and drug resistance similar to daily oral ART [11]. In the LATTE-2 trial, the percentage of participants with HIV-1 RNA suppression through 96 weeks was similar among those who switched to LAI-CAB/RPV and those who continued oral cabotegravir-based therapy [12]. Additionally, as part of these clinical protocols, some studies have quantitatively measured patient self-reported outcomes, such as satisfaction and quality of life, in patients who have transitioned to injectable therapy. In phase 3 ATLAS and FLAIR studies, the investigators utilized patient-reported outcome instruments to measure treatment satisfaction (HIVTSQ) and acceptance (ACCEPT general domain), health status (SF-12), injection tolerability/acceptance (PIN), and treatment preference. In pooled analyses, LAI-CAB/RPV-treated patients demonstrated greater mean improvements from baseline than the oral ART group in treatment satisfaction and acceptance. In both studies, ≥ 97% of long-acting group participants with recorded data preferred LAI-CAB/RPV treatment compared with prior oral therapy [13]. These results further support the potential of a long-acting injectable option for people living with HIV to improve adherence and quality of life. However, it’s important to acknowledge the limitations of quantitative research in capturing the nuanced and multifaceted aspects of patient experiences during this transition. This underscores the need for further qualitative investigations to explore patient perspectives, shedding light on the unique challenges, motivations, and coping strategies individuals employ during this process. This deeper understanding can enhance patient-centered care, provide insights into strategies for improving adherence and treatment outcomes, facilitate discussions with healthcare providers, inform future treatment recommendations, and encourage further research aimed at optimizing long-acting therapies, improving patient outcomes, and developing more patient-friendly treatment options.

A qualitative study was conducted among people living with HIV participating in a Phase II trial of cabotegravir/rilpivirine (LATTE-2) in the United States and Spain [14]. The study explored participant experiences with LAI-CAB/RPV versus daily oral ART. Participants described the convenience of long-acting injections versus daily pills and emotional benefits such as minimized potential for HIV disclosure and eliminating the daily reminder of living with HIV [14]. The study yielded important results, highlighting that participants perceived LAI-CAB/RPV as a highly acceptable and desirable treatment option. This perception stemmed from the convenience of a monthly or bimonthly injection compared to daily pills, as the injectable achieved the benefit of eliminating the daily reminder of living with HIV. Importantly, these positive perceptions were consistent across diverse participant demographics, suggesting that long-acting ART holds promise for a wide range of individuals. This aligns with prior findings on the positive impact of reducing the number of daily pills on adherence and quality of life in HIV-positive patients, underscoring the potential long-term benefits of long-acting ART [14,15]. Nevertheless, the study identified several limitations, including its cross-sectional nature and the sample’s selectivity from a clinical trial, which may have influenced the positive experiences reported by participants. Hence, we aim to comprehensively investigate the treatment experience of individuals living with HIV who have transitioned from conventional oral antiretroviral therapy to long-acting antiretroviral therapy using Cabotegravir/Rilpivirine in Puerto Rico. By exploring the personal narratives of participants, we seek to gain a deeper understanding of their journey, challenges, and successes in adapting to this novel treatment approach.

## Materials and methods

### Study design

We conducted a qualitative study with a phenomenological approach to explore the lived experiences and perspectives of people living with HIV who transitioned from oral ART to LAI-CAB/RPV. Patients who were 21 years old or older, who transitioned from oral ART to LAI-CAB/RPV, and who had been on the injectable for at least six months were invited to be part of the study. Those cognitively impaired that could not provide informed consent were excluded from the study. The research was approved as exempt by the Nova Southeastern University Institutional Review Board on November 15, 2023 (protocol # 2023–576-NSU). This approval was valid and sufficient to conduct our study on our research site, and no additional step was needed.

### Setting

The study was conducted at Centro Ararat, a not-for-profit, Ryan White HIV/AIDS-funded clinic established in 2001, that offers primary health services in Ponce, San Juan, and Arecibo, Puerto Rico to over 1,200 people living with HIV.

Centro Ararat worked the transition from oral ART to long-acting injectable as follows: after medical evaluation, the prescribing physician assesses eligibility criteria for switching to LAI- CAB/RPV. To qualify, patients must have maintained viral suppression for at least six months on their current oral antiretroviral regimen and must have no documented resistance to the prescribed agents. Eligible patients may initiate either an oral lead-in phase or transition directly to the injectable formulation.

Once the decision to initiate LAI-CAB/RPV is made, the physician communicates the treatment plan to the multidisciplinary care team, which includes pharmacy, case management, nursing, and clinical coordinator. The case manager collaborates with the pharmacy department to assess insurance coverage for LAI-CAB/RPV. For patients with private insurance and specialty pharmacy benefits, medication is coordinated through the specialty pharmacy and delivered to the clinic. For those without private coverage, access is facilitated through public programs such as Medicaid or the AIDS Drug Assistance Program.

Upon approval, delivery of the medication is scheduled, and the clinic receptionist coordinates the LAI-CAB/RPV administration appointment. The multidisciplinary team is responsible for ensuring timely receipt of medication every two months and for scheduling administration visits within the recommended timeframe to minimize the risk of delayed dosing and maintain therapeutic continuity. When the study took place, the clinic had only 49 patients who had transitioned to long-acting injectable ART.

### Participant selection

Purposeful sampling was used to recruit study participants. This strategy seeks to gain a deeper understanding of phenomena of interest by selecting rich cases, “from which one can learn a great deal about issues of central importance to the purpose of the research” [16]. Potential participants who were over 21 years old, who transitioned from oral ART to LAI-CAB/RPV, and who had been on the injectable for at least six months were invited to be part of the study. This six-month minimum was chosen since it allows sufficient time for participants to experience both the initial transition and medium-term adaptation to the injectable regimen. It was important to capture not only first impressions but also more sustained reflections on treatment integration into daily life. Study participants that met the inclusion criteria were invited to be part of the study by the staff of Centro Ararat.

Once the participants agreed to be part of the study, an appointment for the face-to-face interview was made. At the moment of the interview, the first author confirmed that the participant indeed met the inclusion criteria and continued with the discussion of the written informed consent. After clarifying doubts and verifying the participant’s understanding, a signature of the informed consent was obtained. Considering the inclusion and exclusion criteria, 10 potential participants were invited to be part of the study and all of them agreed. The interview took place in a private space at Centro Ararat, a familiar and non-threatening environment for the study participants.

### Data collection

Three of the authors developed the question guide: a pharmacy resident, a health educator with experience in qualitative studies and HIV, and a pharmacist specialized in HIV [R.V.P, G.S.S. & K.A.]. R.V.P is a Hispanic male with a pharmacy doctorate degree completing his postgraduate year one pharmacy residency. K.A. is a female pharmacist specialized in HIV with more than 15 years of experience in an HIV ambulatory care setting. G.S.S. is a Hispanic, female researcher with an educational background in public health and who has previously worked in a specialized HIV clinic in Puerto Rico. She has conducted various qualitative research projects and has had formal and informal training in conducting qualitative interviews and focus group. A panel of experts in HIV composed of academic external members and part of the research team reviewed the question guide, assessing its content alignment with the study’s purpose and the questions’ relevancy. The interview guide was later piloted with a potential participant. After the pilot, questions regarding receiving an HIV diagnosis were added. The question guide sought to elicit responses from the participants about their experiences as people living with HIV who transitioned from oral to long-acting injectable ART. Questions about HIV diagnosis, experience with oral ART, and experience and meaning of the transition to LAI-CAB/RPV ART were asked.

Approval from the Nova Southeastern University Institutional Review Board was obtained before conducting the interviews. The first author conducted ten interviews from December 19, 2023, until March 3, 2024. The interviews lasted 30–40 minutes approximately and were voice-recorded and transcribed verbatim. Transcripts were checked for accuracy by the research team. Identifying information was removed from the transcripts to ensure confidentiality. Interviews were conducted in Spanish, the participants’ and researchers’ primary language. All the research files were kept in the principal investigator’s office in a locked cabinet. The voice recordings were transferred to the institutional computer of the first author, which is password protected. The recordings were deleted from the recorder to avoid a breach of confidentiality.

A small sample size in Interpretative Phenomenological Analysis (IPA) is recommended [17].

After completing ten interviews, the research team noted that thematic recurrence was consistent across participants, suggesting that saturation had been achieved. This aligns with the methodological standards of IPA, which prioritizes depth of analysis over large sample sizes.

### Data management and analysis

**Fig 1:**
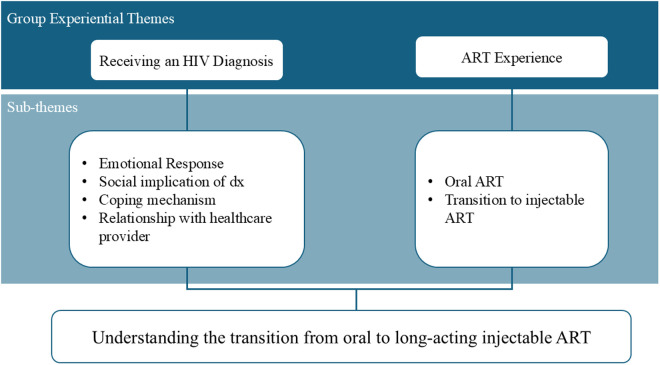
Graphical representation of the data analysis.

Data analysis followed the IPA approach as outlined by Smith, Flowers [17]. The original Spanish interview transcripts were used for data analysis to avoid an additional level of interpretation and ensure accuracy, thereby respecting our participants’ voices. Each transcript was analyzed individually. The process began with multiple readings of the interview transcripts to immerse the researchers in the data and ensure the participants’ voices were central to the analysis. Detailed and comprehensive notes were made, capturing initial thoughts and reflections. These notes were then distilled into experiential statements to reduce the volume of detail while highlighting key points. Connections were mapped to identify patterns and relationships within the data, forming Personal Experiential Themes (PET) for each participant. This process was repeated for all transcripts, ensuring consistency and depth. Finally, the themes were synthesized across all participants to develop Group Experiential Themes (GET), identifying commonalities and differences in their experiences. Two GETs, receiving an HIV diagnosis and ART experience, were identified, along with different sub-themes within each of the GETs. Fig 1 shows a graphic representation of the data analysis. The GET and each sub-theme will be used to illustrate the study findings. English translations of the fragments from participants’ narratives will be included to support each GET and its sub-themes. Two authors (R.V.P. & G.S.S.), knowledgeable in English and Spanish and the Puerto Rican culture translated the finals quotes added in this manuscript. Study participants selected the fictional name used in this manuscript to protect their identity.

Data analysis was conducted manually by three members of the research team (R.V.P., G.S.S. & F.B.). No codes were established a priori. Each of the coders worked independently, and after coding all the interviews a final meeting took place to discuss individual analysis and discuss discrepancies. When facing discrepancies in the coding, a discussion took place until consensus was reached. Anonymized excerpts of interview transcripts may be made available upon reasonable request to the corresponding author, subject to IRB approval and participant confidentiality agreements.

## Findings

### Participant demographics and characteristics

The study included 10 participants living with HIV who transitioned from oral ART to LAI-CAB/RPV. Participants’ mean age was 67 years (SD = 9), ranging from 46 to 81 years (see Table 1). Seventy percent of the study participants were men, and 30% were women. Most of them identify themselves as heterosexual (60%) and 40% as homosexual. On average, study participants have taken oral ART medication for 23.2 years (SD = 8.7), ranging from 10 to over 30 years. Study participants have been on LAI-CAB/RPV for at least 6–24 months. All participants had a viral load of ≤ 50 cp/mL, with CD4 counts within the normal range, which were maintained both before and after the transition to the injectable therapy. Common comorbidities included hyperlipidemia (70%), hypertension (60%), hypothyroidism (40%), and diabetes mellitus type 2 (30%).

**Table 1:** Participants baseline characteristics.

Characteristics	n (%)	
**Health insurance:** Private health Insurance Medicaid Medicare	3 (30%) 8 (80%) 6 (60%)	
**Years in oral ART:** 10-15 16-20 21-25 26-30 ≥ 30	3 (30%) 1 (10%) 1 (10%) 4 (40%) 1 (10%)	
**Months in LAI-CAB/RPV:** 6-11 12-23 ≥ 24	1 (10%) 4 (40%) 5 (50%)	
**Comorbidities:**		
Hyperlipidemia Hypertension Hypothyroidism ^*^DM-2 ^*^GERD Psychiatric Disorders Sleep Disorders ^*^COPD ^*^ESRD ^*^CKD Coronary artery disease Congestive Heart Failure Anemia Diabetes type I Endometriosis Cervicalgia Osteoarthritis Atopic dermatitis Dysphagia	7 (70%) 6 (60%) 4 (40%) 3 (30%) 3 (30%) 2 (20%) 2 (20%) 1 (10%) 1 (10%) 1 (10%) 1 (10%) 1 (10%) 1 (10%) 1 (10%) 1 (10%) 1 (10%) 1 (10%) 1 (10%) 1 (10%)	
**Quantity of current daily oral medications:**		
None 1-3 Medications 4-6 Medications 7-9 Medications	1 (10%) 2 (20%) 5 (50%) 2 (20%)	
**Viral load:**		
≤ 50 cp/mL	10 (100%)	
^*^ **CD4 Count:**	Before transitioning	After transitioning
250-499 cells/µL 500-999 cells/µL 1000-1499 cells/µL ≥ 1500 cells/µL	5 (50%) 3 (40%) 1 (10%) 1 (10%)	4 (40%) 4 (40%) 0 (0%) 2 (20%)

* DM-2, diabetes mellitus-2; GERD, gastroesophageal reflux disease; COPD, chronic obstructive pulmonary disease; ESRD, end-stage renal disease; CKD, chronic kidney disease; CD4, cluster of differentiation 4.

### Experience with oral ART

For most of our study participants, receiving an HIV diagnosis was a life-changing event, as Angélica, a 46-year-old female, shared, *“It felt like the ground disappeared beneath my feet. I thought my life was over.”* In addition to having to manage the implications of their new HIV diagnosis, they learned that they needed to take ART medication for the rest of their lives. They later understood that their ART was essential for their lives but did come with challenges and struggles. In this section, you will get to know our participants’ lived experiences with their oral regimen.

Participants’ experiences with oral ART were marked by several challenges, including the complexity of regimens, adverse effects, and social implications. Many started with regimens involving multiple medications taken several times a day. Dennis, a 65-year-old male, recalled

*“Back when I started... with oral medication, cocktails still existed. It was a cocktail of 10 to 15 pills morning, noon, and night. So how was I supposed to socialize publicly at work and all that having to gulp down those cocktails’ morning, noon, and night?”*

Adverse effects ranged from gastrointestinal discomfort to psychological distress. Reiki, a 68-year-old male, recounted, “*There was one now I can’t remember the name that raised my liver enzymes a lot. They had to suspend the treatment. They raised them almost to a thousand”.* Lipodystrophy had a profound psychological impact. July, a 64-year-old male, noted, *“...and I’ll always remember that when the side effects started like lipodystrophy that really affected me emotionally”.*

Adherence challenges were prominent, including the burden of daily medication, difficulties swallowing large pills, and disruptions to daily life. Ernesto, a 61-year-old male, emphasized the necessity of adherence, saying, “*For me, my life depends on those bottles. If I don’t take them, I will die”.* Participants often had to hide their medications to avoid stigma and maintain privacy. Laura mentioned, *“I used to hide my pills in the fridge so that my family wouldn’t find out”.*

The social implications of oral ART were significant. The need to take multiple doses throughout the day often disrupt social activities, travel plans, and work commitments, leading to feelings of isolation and frustration. For instance, Reiki shared,

*“...at that moment it made me nervous passing through customs or airport security seeing a young person with so many pill bottles because the medications were three huge bottles. Plus, with that concern of having to carry enough supply of the medications for every day, it creates tension”.*

### Transition to LAI-CAB/RPV

Our study participants considered the transition to LAI-CAB/RPV a transformative experience that impacted their lives from many standpoints. All of them considered LAI-CAB/RPV something positive that improved their lives personally and socially and helped them have a sense of ‘normalcy’.

Study participants learned about LAI-CAB/RPV through different sources, such as leaflets, at the pharmacy, and through a talk by the clinic’s director. The idea of a new long-acting injectable treatment for HIV was well received by the study participants. As Laura shared:

*[referring to LAI-CAB/RPV] “I already knew it was in the making; I have been waiting for 15 years and said yes. I use it every month; I don’t care. If you prick me, I don’t care. And she [referring to the clinic staff] asks me, are you sure? And I said, yes, I am”.*

Most of the study participants were invited to transition to the LAI by the healthcare provider at Centro Ararat and access to the injectable therapy was facilitated by them who efficiently managed the paperwork and approval process. Ernesto highlighted the ease of accessing injectable therapy: *“Yes, that was a blessing... when the doctor approached me, I said yes, let’s see if the medical plan approves it, and thanks to the Lord, I haven’t had any kind of situation”.* However, some faced delays with pharmacy services, requiring proactive follow-up to ensure timely delivery, as mentioned by July:

“*I mean the bureaucratic process of medical plans and the specialized pharmacy that is dedicated to distributing the medication... I have to keep pushing, as one says, the specialized pharmacy to ensure it arrives on time because it is very important for me, and I told them so... I mean, every two months I have to stay on top of it, and it creates tension for me”.*

The first-dose experience elicited mixed emotions of excitement and anxiety. Ernesto described his anticipation as a *“gift of life,”* while also noting initial concerns about potential side effects and reactions. Laura expressed a similar sentiment, feeling both nervous and hopeful about the new treatment. Ismael recounted his positive feelings, stating, “*I felt happy because it’s a success. It’s every two months that I don’t have to worry about pills anymore”.*

Most participants reported mild discomfort at the injection site, which subsided within a day. Some experienced mild symptoms such as body aches or chills, manageable with over-the-counter medication. Angélica, reflecting on her experience, stated:

*“I do feel a little sore in the area if I touch it and such, but it wasn’t something like I couldn’t walk. But I didn’t mind as long as I don’t have to take a pill anymore, I’m happy”.*

Participants demonstrated a high level of acceptance and adaptability to potential adverse effects, emphasizing their willingness to tolerate discomfort in exchange for the benefits of injectable therapy.

Participants showed a remarkable level of resilience and adaptability to the new regimen. Ismael noted, *“I didn’t have any reactions to the medication. I felt good, thank God*.” Laura highlighted the improvement in her quality of life, stating, *“The first injection was a bit nerve-wracking, but after that, everything improved so much. I told my doctor he saved my life”.*

Participants demonstrated high adherence to LAI-CAB/RPV, appreciating the reduced frequency of clinic visits and the flexibility it afforded. Laura mentioned, *“Before, I had to come monthly for medication refills. Now, I only come every two months, which is much more convenient”.* Participants also noted the ease of incorporating the bi-monthly clinic visits into their routines without the daily reminder of their condition, as was the case with oral ART.

**Fig 2:**
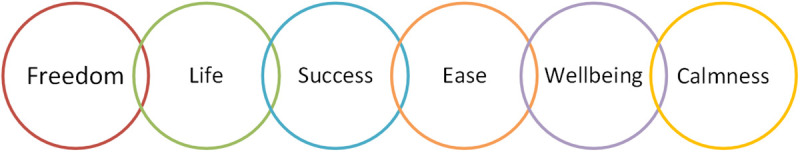
Key terms used by participants to describe the meaning of this experience.

The transition to LAI-CAB/RPV brought significant emotional relief and a sense of normalcy. Participants emphasized freedom from the constant reminder of their condition that daily pills represented. Angélica described it as, *“Freedom. It means freedom. I don’t have to think, did I take it, did I not take it, where is the alarm? It’s like normalizing my life”.* Laura echoed this sentiment, stating that the injectable therapy provided her with more security and optimism for the future. See Fig 2 to see other terms used to describe the meaning participants ascribed to LAI-CAB/RPV.

Participants also experienced a positive impact on their social lives. The reduced burden of medication allowed for more spontaneous and less restricted social interactions. Laura highlighted the transformative effect, saying, *“I feel more like any other person now. I get the injection, and then I don’t have to think about it for two months”.*

The role of healthcare providers was crucial in the successful transition to LAI-CAB/RPV. Participants expressed gratitude for the support and guidance provided by their doctors and clinic staff. Ernesto highlighted the seamless process facilitated by his healthcare team, *“The doctor and pharmacist coordinated everything. I didn’t have any issues with approval, and the process was smooth*.” Laura also praised her healthcare providers, noting, *“The staff here are excellent. They made sure everything was taken care of, and I felt supported throughout the transition”.*

The transition to LAI-CAB/RPV had a profound impact on participants’ quality of life. The convenience of reduced medication burden, improved adherence, and enhanced emotional well-being were consistently highlighted. Angélica summed up her experience, *“It’s been a huge relief. I don’t have to worry about taking pills every day, and I feel more at peace”.*

Participants reported a significant improvement in their overall well-being, both physically and emotionally. Laura mentioned, “*Emotionally, I feel much better. I don’t have the daily reminder of my condition, and that has made a big difference in my life”.* Ismael shared a similar experience, noting the positive impact on his mental health, *“I feel much better since using the injectable. I don’t have the constant worry about my medication, and that has improved my emotional state”.*

Participants were asked to provide recommendations for others considering the transition to LAI-CAB/RPV. The advice was overwhelmingly positive, with many encouraging others to make the switch. Angélica advised, “*Don’t be afraid. It’s much easier than taking pills every day, and it has made a big difference in my life.”* Laura emphasized the importance of staying informed and communicating with healthcare providers, *“Seek information, talk to your doctor, and don’t be afraid to ask questions. This treatment has been a game-changer for me”.* Participants also highlighted the importance of support from healthcare providers and the value of patient education. Ernesto suggested, *“Make sure your healthcare team is supportive and that you understand the process. The transition can be smooth if you have the right information and support”*.

## Discussion

The transition from oral ART to LAI-CAB/RPV significantly improved the medication experience among our study participants. This finding aligns with existing literature indicating that long-acting injectables can reduce the daily medication burden, leading to better adherence and medication experience [10,18]. A study by Orkin, Arasteh [19] demonstrated that LAI-CAB/RPV was non-inferior to daily oral ART in maintaining viral suppression, highlighting its efficacy and potential to address adherence challenges [20].

A key advantage of LAI-CAB/RPV is the reduced frequency of dosing, which mitigates the common barriers associated with daily oral regimens. Our participants reported high satisfaction with the bi-monthly injections, echoing findings from the National Institutes of Health which noted that long-acting ART is particularly beneficial for individuals who struggle with the daily discipline required for oral ART [21]. This is further supported by research showing that long-acting injectables can significantly enhance the quality of life by eliminating the daily reminder of the illness, thus reducing stress and improving mental health [20].

The improvement in quality of life reported by our participants is consistent with the broader research landscape. A study highlighted that patients on long-acting injectables experienced significant improvements in their overall well-being and satisfaction compared to those on daily oral regimens [10]. The reduction in pill burden and the increased convenience of less frequent dosing were major factors contributing to this enhanced quality of life.

Moreover, the emotional relief from not having to take daily medication can have profound psychological benefits. Participants in our study, like Angélica, who described feeling a sense of “freedom” from daily pills, mirror sentiments expressed in other studies. For instance, research found that the majority of participants favored long-acting injectables due to the mental and emotional relief from daily medication routines [14,22].

The adaptability of our participants to minor adverse effects from LAI-CAB/RPV is well-documented in clinical trials. The studies indicate that while injection site reactions are common, they are generally mild and manageable [10,18]. Our participants’ experiences align with these findings, showing that the benefits of reduced medication burden outweigh the occasional discomfort of injections.

Furthermore, the broader acceptance of LAI-CAB/RPV despite these minor side effects underscores the importance of considering patient preferences in treatment plans. As highlighted in the European Medicines Agency’s report, long-acting injectables offer a significant improvement in treatment satisfaction and adherence, which are crucial for the long-term management of HIV [20].

Stigma remains a significant barrier to effective HIV treatment, as evidenced by the reluctance of our participants to disclose their status. This is consistent with findings in the literature that stigma negatively impacts mental health and social support networks [14]. For many individuals living with HIV, medication is a lifesaving thing, but it also implies a social vulnerability since it puts them at risk of revealing their diagnosis. In a qualitative study conducted in Puerto Rico, stigma played a key role in medication intake. A study participant was reluctant to take their medication at work since she fears others knowing her HIV diagnosis. Other reported stopping their medication intake due to being rejected and stigmatized by others [23] The transition to LAI-CAB/RPV, by reducing the visible signs of medication intake and thus the potential for stigma, can alleviate some of these social burdens. The reduced need for daily medication can help individuals manage their condition more discreetly, thereby reducing the fear of disclosure and associated stigma.

Comparing our findings with those related to oral ART, the advantages of LAI-CAB/RPV become evident. Daily oral regimens, despite their effectiveness, are often associated with various challenges, including regimen complexity, adverse effects, and social inconvenience [24]. Our study participants’ experiences with oral ART, characterized by difficulties in maintaining adherence and managing side effects, reflect these widespread issues. Long-acting injectables, by offering a less burdensome alternative, address many of these challenges. Research has consistently shown that patients on long-acting injectables report fewer adherence issues and a higher quality of life compared to those on daily oral ART [18,19]. This aligns with our participants’ reports of improved medication experience and satisfaction with LAI-CAB/RPV.

The positive outcomes associated with LAI-CAB/RPV in our study have significant implications for clinical practice. Healthcare providers should consider the benefits of long-acting injectables, particularly for patients who face challenges with daily oral regimes. The findings from our study, supported by extensive research, suggest that LAI-CAB/RPV can enhance adherence, reduce stigma, and improve overall quality of life for people living with HIV.

Implementing long-acting injectables more broadly could address persistent non-adherence issues and improve clinical outcomes. The World Health Organization (WHO) has emphasized the potential of long-acting injectables in reducing the global burden of HIV by improving treatment adherence and outcomes [25]. In a brief report, LAI-CAB/RPV showed promising results in 12 patients with viremia who were initiated directly on monthly injectable therapy. All patients achieved viral suppression (<50 copies/mL) in three months with no virological rebound [26]. These are promising findings for people living with HIV who struggled to achieve and maintain viral suppression with traditional oral therapies. It also highlights the potential for improved patient care and management strategies in the treatment of HIV.

Our qualitative findings align closely with the outcomes of key quantitative trials, participants in those studies reported high levels of treatment satisfaction, and in both ATLAS and FLAIR, ≥ 97% of participants preferred the injectable over oral therapy [13]. Our participants echoed these preferences, highlighting the emotional relief and improved quality of life associated with reduced pill burden and fewer reminders of HIV. This qualitative dimension adds depth to the numerical outcomes, providing context to the high adherence and preference rates observed in the trials. Furthermore, our findings support international policy recommendations.

The European Medicines Agency has endorsed LAI-CAB/RPV as a viable alternative for virologically suppressed individuals, citing its potential to improve treatment satisfaction and reduce stigma [20]. Similarly, the WHO recommends long-acting formulations as part of a broader strategy to improve HIV care delivery and overcome adherence barriers [25]. Our data reinforces these policies by demonstrating real-world patient experiences that validate the psychosocial and clinical benefits anticipated in regulatory and policy frameworks.

According to our study, participants’ access to LAI-CAB/RPV was greatly facilitated by supportive healthcare providers, which contributed to a seamless care experience. Effective communication with providers was identified as a key factor that enhanced patient satisfaction and ensured the timely administration of the medication.

To optimize the LAI-CAB/RPV experience, it is critical to establish a formalized workflow that minimizes the risk of delays in medication administration. Although certain factors, such as delays in drug shipment or transportation, may be outside the clinic’s control, the recommended 7-day administration window provides valuable flexibility. Ongoing communication among patients, healthcare providers, case managers, and dispensing pharmacies is essential to maintaining continuity of care.

Comprehensive provider training is also a fundamental component of successful LAI-CAB/RPV implementation and should be incorporated into the clinic infrastructure prior to service rollout. The U.S. Department of Health and Human Services guidelines emphasize the importance of clinical staff proficiency in intramuscular injection techniques, including site selection and needle length, particularly for patients with a body mass index greater than 30 kg/m² [27]. Training should also address the management of injection site reactions and post-injection discomfort. Furthermore, staff should be equipped to manage missed doses, utilize oral bridging therapy when necessary, and identify potential drug-drug interactions relevant to LAI-CAB/RPV therapy.

The limitations of the study were as follows: At the time of the study, the availability of LAI-CAB/RPV was very limited in Puerto Rico. In addition, patients who receive care in this clinic come from multiple municipalities on the island of Puerto Rico expanding the scope of this study beyond the physical location of the clinic. Most of our participants were older adults, which may not fully capture the experiences of younger individuals living with HIV. Additionally, the participants received care from a specialized HIV clinic, which could have positively influenced their experience with the injectable therapy. Despite these limitations, the age diversity of our participants allows for valuable insights into the long-term management of HIV and the unique challenges faced by older adults. The older age of our participants provides insightful experiences of the success of transition to LAI-CAB/RPV in this age group, some with decades of traditional oral therapies. Furthermore, the high-quality care provided by the specialized clinic underscores the potential benefits of comprehensive, patient-centered care in optimizing treatment outcomes. These strengths highlight the importance of continued support and tailored interventions to enhance the quality of life for people living with HIV across different age groups and care settings.

## Conclusion

The transition from oral ART to LAI-CAB/RPV in our study participants has demonstrated substantial benefits, including improved medication experience, enhanced quality of life, and reduced stigma. These findings are consistent with broader research, underscoring the potential of long-acting injectables to address the challenges associated with daily oral ART and to revolutionize the standard of care for people living with HIV. Future research and clinical practice should continue to explore the use and expand access to long-acting injectable ART to maximize its benefits for diverse populations living with HIV.
